# PDE3 inhibitor and EGCG combination treatment suppress cancer stem cell properties in pancreatic ductal adenocarcinoma

**DOI:** 10.1038/s41598-017-02162-9

**Published:** 2017-05-15

**Authors:** Motofumi Kumazoe, Mika Takai, Shun Hiroi, Chieri Takeuchi, Maasa Yamanouchi, Takashi Nojiri, Hiroaki Onda, Jaehoon Bae, Yuhui Huang, Kanako Takamatsu, Shuya Yamashita, Shuhei Yamada, Kenji Kangawa, Takashi Takahashi, Hiroshi Tanaka, Hirofumi Tachibana

**Affiliations:** 1 Division of Applied Biological Chemistry, Department of Bioscience and Biotechnology, Faculty of Agriculture, Kyushu University, 6-10-1 Hakozaki, Higashi-ku, Fukuoka 812-8581 Japan; 2 Department of Applied Chemistry, Graduate School of Science and Engineering, Tokyo Institute of Technology, 2-12-1 Ookayama, Meguro, Tokyo 152-8552 Japan; 3 Department of Biochemistry, National Cerebral and Cardiovascular Center Research Institute, 5-7-1 Fujishiro-dai, Suita-City, Osaka 565-8565 Japan; 4 Yokohama College of Pharmacy 601, Matana-cho, Totsuka-ku, Yokohama, Kanagawa 245-0066 Japan

## Abstract

Recurrence following chemotherapy is observed in the majority of patients with pancreatic ductal adenocarcinoma (PDAC). Recent studies suggest that cancer stem cells (CSCs) may be involved in PDAC recurrence and metastasis. However, an efficient approach to targeting pancreatic CSCs remains to be established. Here we show that in cancer cells overexpressing the 67-kDa laminin receptor (67LR)-dependent cyclic GMP (cGMP) inducer, epigallocatechin-3-*O*-gallate (EGCG) and a phosphodiesterase 3 (PDE3) inhibitor in combination significantly suppressed the Forkhead box O3 and CD44 axis, which is indispensable for the CSC properties of PDAC. We confirmed that the EGCG and PDE3 inhibitor in combination strongly suppressed tumour formation and liver metastasis *in vivo*. We also found that a synthesized EGCG analog capable of inducing strong cGMP production drastically suppressed the CSC properties of PDAC and extended the survival period *in vivo*. In conclusion, the combination treatment of EGCG and a PDE3 inhibitor as a strong cGMP inducer could be a potential treatment candidate for the eradication of CSCs of PDAC.

## Introduction

Pancreatic ductal adenocarcinoma (PDAC) is a critical cancer with the worst prognosis. Recent cancer statistics indicate that PDAC is the fourth most frequent type of malignancy-caused mortality in developed countries^1^. Due to the lack of characteristic symptoms and an efficient established method for the early detection of PDAC, the majority of patients with PDAC are diagnosed at an advanced stage^1^. Considering the difficulty to identify PDAC early, the establishment of an efficient systemic chemotherapy is in high demand.

Several advances have been achieved regarding chemotherapy for PDAC, including gemcitabine^2^ and FOLFIRINOX, a chemotherapy regimen consisting of fluorouracil, leucovorin, irinotecan and oxaliplatin^3^. However, the five-year survival rate has not changed by more than approximately 5% during the past 40 years and the majority of patients (approximately 95%) experience a recurrence following chemotherapy^4^. Considering these situation, the establishment of a novel therapeutic strategy is particular importance.

Recently, a small subset of pancreatic cancer stem cells (CSCs) has been shown to be resistant to radiation and chemotherapy^4^. In this context, reports suggest that CSCs are responsible for tumor initiation, progression and metastasis^5, 6^. Emerging evidence indicates that Hedgehog, Wnt/β-catenin and the Notch signalling pathways play a crucial role in PDAC^7, 8^. However, these signaling cascades are also highly involved in the maintenance of normal somatic stem cells and targeting these pathways may cause critical adverse effects^9, 10^. Moreover, clinical study found that a Hedgehog inhibitor could not improve the prognosis of patients with PDAC^11^. Thus, an efficacious approach for targeting the CSCs of PDAC has yet to be established.

Cyclic GMP (cGMP) is a well-known second messenger produced by soluble guanylyl cyclases (sGC) in response to nitric oxide (NO) and by natriuretic peptide receptor-A/B in response to natriuretic peptides (e.g. atrial natriuretic peptide)^12^. cGMP is involved in the homeostasis of blood vessels and sexual arousal-induced penile erection^13^. Recently, we revealed that cGMP has an inhibitory effect on pancreatic CSC properties^14^. By studying the mechanisms of the inhibitory effect of cGMP on CSCs, we revealed that Forkhead box O3 (FOXO3)/CD44 axis inhibition by cGMP plays a central role in its unique effect^14^. Importantly, systemic FoxO3 KO mice were found to be normal and did not exhibit any statistically significant differences in mortality compared with wild-type littermates for up to 48 weeks of age^15^.

The 67-kDa laminin receptor (67LR), known as the 37-kDa oncofetal antigen is overexpressed in several types of cancers, including multiple myelomas, gastric cancer, prostate cancer and pancreatic cancer^16–19^. Pathological studies suggest that the increased expression of 67LR is associated with the risk of tumor progression^18, 19^. In cancer cells, the abnormal overexpression of 67LR induced the enhancement of metastasis and tumor growth through the overexpression of both cyclin-dependent kinases 1/2 and cyclins (A and B)^20^. Furthermore, 67LR induces an acquired chemotherapeutic drug-resistant phenotype^21^. Collectively, 67LR has an essential role in the development and maintenance of cancer.

We previously identified that 67LR acts as the receptor of (–)-epigallocatechin-3-*O*-gallate (EGCG), the major green tea polyphenol^17, 22^. Surprisingly, the Akt, endothelial nitric oxide synthase (eNOS), cGMP signaling axis elicited by EGCG bound to 67LR has a crucial role in the anti-cancer effect of EGCG^17^. We and others have shown that this compound kills 67LR-overexpressing cancers without affecting normal cells^16, 17, 23^.

Phosphodiesterases (PDEs) consist of any enzyme that breaks a phosphodiester bond of cGMP or cAMP. The characteristic isoforms of PDEs modulate different signaling pathways and provide ideal drug targets^24, 25^. Indeed, several PDE inhibitors are widely used for treatment. Recently, some types of PDEs have been found to be overexpressed in cancer cells and provide another potential target for treatment^17, 26, 27^. However, little is known about the expression levels of PDE3.

In the present study, we demonstrate that PDE3A was overexpressed in PDACs especially, CD44^+^ cells. A therapeutic regimen combining a PDE3 inhibitor and a cancer-overexpressed 67LR-dependent cGMP inducer significantly suppressed CSC properties *in vitro*. We also confirmed that this combination suppressed PDAC tumor growth and metastasis *in vivo*. Moreover, an EGCG analog with strong cGMP induction properties extends the survival time in a mouse xenograft model. Taken together, our data suggest that the 67LR-dependent cGMP inducer and PDE3 inhibitor in combination or an EGCG analog with strong cGMP induction properties could be a potent candidate for PDAC therapy.

## Results

### PDE3 overexpression in CD44^+^ cells of PDAC

**Figure 1:**
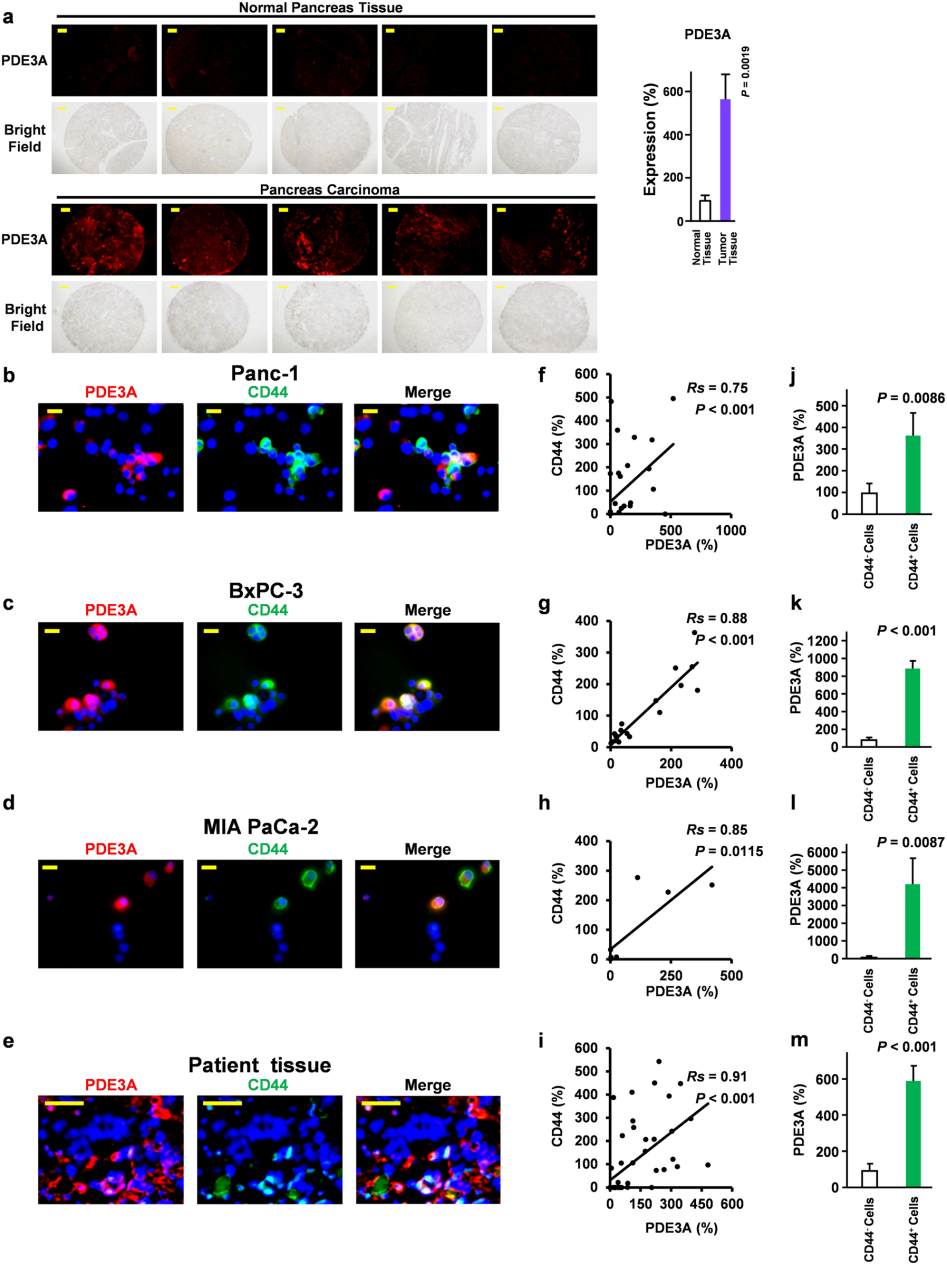
PDE3A was overexpressed in CD44^+^ cells of PDAC. (**a**) Immunofluorescence staining for PDE3A of pancreatic cancer patient tissues (×10) (*n* = 5). (**b**–**e**) Panc-1, BxPC-3, and MIAPaCa-2 cells (×40), PDAC patient tissues (×60). (**f**–**i**) Correlation between CD44 expression and PDE3A expression in cells (Panc-1 cells *n* = 32, BxPC-3 cells *n* = 19, MIA PaCa-2 cells *n* = 8, Primary cancer cells *n* = 53). (**j**–**m**) Expression of PDE3A in CD44^−^ cells and CD44^+^ cells ((**j**) CD44^−^ cells *n* = 21, CD44^+^ cells *n* = 11, (**k**) CD44^−^ cells *n* = 12, CD44^+^ cells *n* = 7, (**l**) CD44^−^ cells *n* = 5, CD44^+^ cells *n* = 3, (**m**) CD44^−^ cells *n* = 34, CD44^+^ cells *n* = 19). A Spearman rank test was used for correlations. All data are presented as means ± SEM.

cGMP is a known messenger involved in vascular homeostasis. The direct or indirect approach to increase cGMP levels is a well-established approach used for the relief of several diseases, including cardiovascular disease and erectile dysfunction^13^. The use of a PDE inhibitor is one of the major approaches to increase cGMP levels indirectly^12^. We previously reported that cGMP has an inhibitory effect on the CSC properties in PDAC^14^ and a clinical study showed PDE3 inhibitors to be safe in patients with cancer and are beneficial for the prevention of atrial fibrillation^28^. These findings suggest that PDE3 inhibition could be potentential target candidate for PDAC and thus, we first assessed the expression levels of PDE3 in PDAC patient tissues. Our immunofluorescence analysis revealed that the expression levels of PDE3A were highly increased (approximately 400% increase, P = 0.0019) compared with normal counterparts (Fig. 1a).

In multiple types of cancers, including breast, colon, prostate and pancreatic cancer, CD44 is identified as a marker of CSCs^8^. Recently, CD44 has been identified as both a marker of CSCs, as well as but also act as a master regulator of CSCs^29^. Moreover, a CD44 knock-in is sufficient to induce CSC properties^29^. We also demonstrated that the silencing of CD44 is sufficient to deprive PDAC cells of CSC properties^14^. Since cGMP can suppress CSC properties^14^, we hypothesized that PDE3A is overexpressed in CD44^+^ cells. Our data indicate that PDE3A is strongly expressed in CD44^+^ cells of three different PDAC cell lines, Panc-1, BxPC-3 and MIA PaCa-2 (Fig. 1b–d). We also demonstrated that PDE3A was strongly overexpressed in CD44^+^ cells in the tumor tissues of PDAC patients (Fig. 1e). Moreover, there was a significant correlation between the expression level of CD44 and PDE3A in the PDAC cell lines Panc-1, BxPC-3 and MIA PaCa-2 (Fig. 1f–h), as well as the tumor tissues derived from PDAC patients (Fig. 1i). Furthermore, we confirmed that PDE3A is significantly overexpressed in CD44^+^ cells compared with CD44^−^ cells (Fig. 1j–m). We also assessed the expression of PDE3A in CD44^+^ cells by flow cytometry analysis. Our data suggested that PDE3A are highly overexpressed in CD44^+^ cells (Supplementary Figure 1a). Collectively, PDE3A was strongly expressed in CD44^+^ cells in PDAC. Further, our results showed that PDE3s are expressed in Panc-1 cells (Supplementary Figure 1b). Our previous clinical study based on 40 patients showed that the PDE3 inhibitor was safe even among patients with heart diseases who presented elevated preoperative BNP levels (≥30 pg/mL)^28^ while few clinical studies have provided information on the safety of the other PDE inhibitors administration for patients with cancer.

### PDE3 inhibitor synergistically potentiated the inhibitory effect of EGCG on CSCs

**Figure 2:**
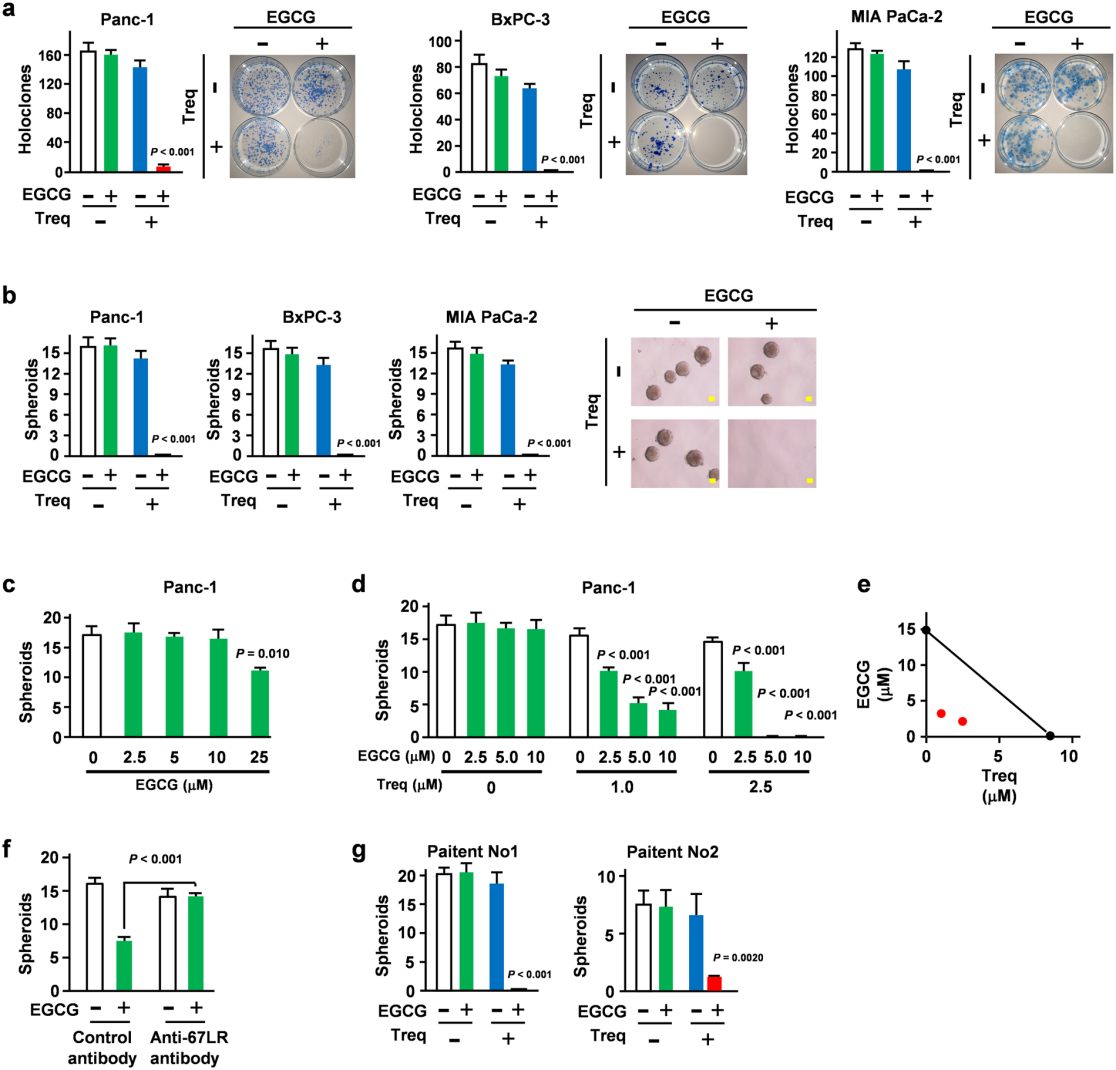
PDE3 inhibitor synergistically potentiated the inhibitory effect of EGCG on CSCs. (**a**) Colony assay in the presence or absence of EGCG (5 μM) or trequinsin (2.5 μM) (*n* = 3). (**b–d**) Spheroid assay in the presence or absence of EGCG or trequinsin (2.5 μM) (*n* = 3). Scale bar: 50 μm. (**e**) Isobologram analysis revealed the synergism of EGCG plus trequinsin combination. (**f**) Panc-1 cells were pretreated with anti-67LR antibodies or control IgM antibodies for 3 h, then treated with EGCG (25 μM) for 21 days (*n* = 3). (**g**) Spheroid assay in the presence or absence of EGCG or trequinsin (*n* = 3). All data are presented as the means ± SEM.

Polyphenon E™ is a 60% EGCG-containing drug that has been approved for relief of the patients with perianal and external genital warts by the US FDA^30^. Recently, Phase II clinical trial involving cancer patients found that Polyphenon E™ was well tolerated and that 29 of 42 patients with chronic lymphocytic leukaemia (69%) exhibited evidence of a biological response, including decreased lymphadenopathy^31^. We previously identified cGMP as the second messenger that mediates the EGCG-induced 67LR-dependent signal^17^. In that paper, we confirmed that the EGCG-induced cGMP induction was completely canceled by anti-67LR antibody pretreatment. We also confirmed that EGCG induced the activation of Akt and induced eNOS phosphorylation at Ser1177, all well-known upstream mechanisms involved in cGMP production through 67LR. Our study showed that a high dose of EGCG could increase cGMP level in pancreatic cancer (Supplementary Figure 2). Since both 67LR (the target of EGCG) and PDE3 (the negative regulator of cGMP) are overexpressed in PDAC, we hypothesized that targeting cancer overexpressing both 67LR and PDE3 could be an applicable approach to suppress the CSC properties of PDAC. Colony and spheroid formation are the characteristic properties of CSCs^32^. We then determined the effect of the combined treatment of EGCG (5 μM) and the PDE3 inhibitor, trequinsin (2.5 μM), on colony formation of three different PDAC cell lines, including Panc-1, BxPC-3 and MIA PaCa-2 cells. Our data suggest that 5 μM of EGCG, the same concentration as the plasma concentration previously observed in clinical trials^33^ could not suppress the colony formation of the PDAC cell lines (Fig. 2a). However, in the presence of the PDE3 inhibitor, EGCG drastically suppressed the colony formation of all PDAC cell lines (Fig. 2a). Consistent with those findings, the combination of the PDE3 inhibitor and EGCG also suppressed the spheroid formation of these PDAC cell lines (Fig. 2b).

To evaluate the impact of the pharmacological inhibition of PDE3 on the inhibitory effects of EGCG, we performed an isobologram analysis, a widely used method for assessing the synergism of two different compounds through plotting the IC_50_ dose of each drug alone and in combination^34^. The inhibitory effect of EGCG on spheroid formation in Panc-1 cells was very limited (Fig. 2c). In contrast, the pretreatment with 2.5 μM PDE3 inhibitor trequinsin drastically increased the inhibitory effect of EGCG on the CSC properties of Panc-1 cells which decreased the IC_50_ dose from 14.8 μM to 2.1 μM (Fig. 2d). An isobologram analysis based on the inhibition of spheroid formation in Panc-1 cells suggested that that combination of the PDE3 inhibitor trequinsin and EGCG was greater than an additive effect (Fig. 2e). We also confirmed that the inhibitory effect on the spheroid formation by EGCG can be blocked by pretreatment with an anti-67LR antibody (Fig. 2f). We further confirmed that the PDE3 inhibitor significantly enhanced the inhibitory effect of EGCG on the spheroid formation of two different primary PDAC cells derived from two different patients (Fig. 2g). Collectively, the combination of the PDE3 inhibitor and EGCG drastically suppressed the CSC properties of PDAC cells.

**Figure 3:**
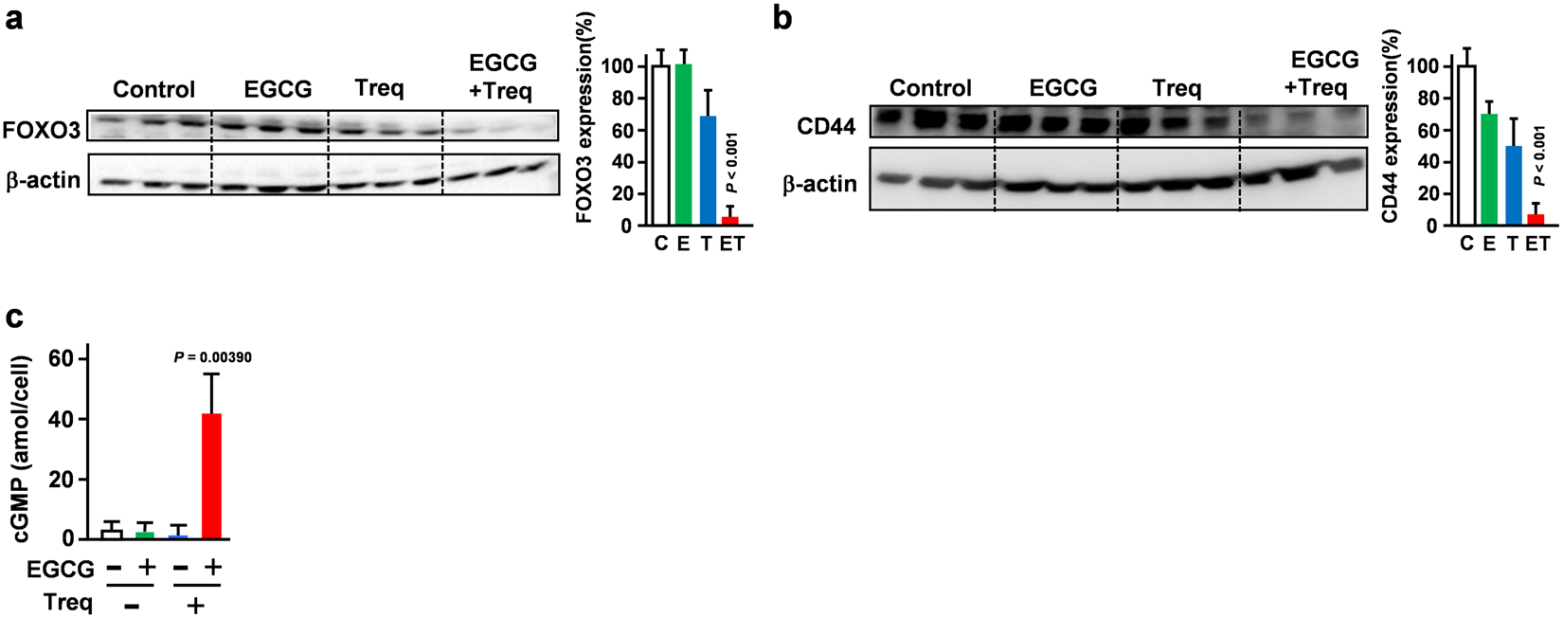
PDE3 inhibitor synergistically potentiated the inhibitory effect of EGCG on the FOXO3 and CD44 axis. (**a**) FOXO3 (48 h) and (**b**) CD44 (72 h) levels measured by Western blotting after EGCG (5 μM) and trequinsin (2.5 μM) treatment in Panc-1 cells. (*n* = 3). (**c**) Intercellular cGMP level measured after treatment with EGCG (5 μM) and the PDE3 inhibitor, trequinsin (2.5 μM) for 3 h (*n* = 3).

We previously reported that cGMP induction strongly suppressed cancer stem cell properties though suppression of FOXO3/CD44 axis^14^. In that study, our microarray analysis showed that inhibition of FOXO3 is the main mechanism of the inhibitory effect of cGMP on both CD44 expression and CSC properties. To assess the effect of the combined treatment of the PDE3 inhibitor and EGCG on the expression of FOXO3 in PDAC cells, Panc-1 cells were treated with EGCG (5 μM) and the PDE3 inhibitor trequinsin (2.5 μM) in combination. Our immunoblot analysis revealed that the combination treatment significantly suppressed FOXO3 expression, the target gene of cGMP (Fig. 3a). This is consistent with the finding that this combination also suppressed CD44 expression, the master regulator of CSC in PDAC cells (Fig. 3b) and drastically increased intracellular cGMP levels (Fig. 3c). Our results also suggested that removing these drugs resulted in the attenuation of the inhibitory effect of CSC properties (Supplementary Figure 3a–c). We performed experiments to confirm whether the combination had a specific effect on CSCs or a general toxic effect. Our data showed that erlotinib (25 μM) and oxaliplatin (25 μM) showed similar or stronger cancer proliferation suppressing- and apoptosis-inducing effects compared with the EGCG/PDE3 inhibitor combination (Supplementary Figure 4a,b). In contrast, the anti-cancer agents erlotinib (25 μM) and oxaliplatin (25 μM), at doses sufficient to elicit the impairment of progenitor cell proliferation (Supplementary Figure 4a,b) did not suppress colony or spheroid formation (Supplementary Figure 4c,d). We also confirmed that these anti-cancer agents did not suppress the FOXO3/CD44 axis (Supplementary Figure 4e). Our results suggested that the impairment of progenitor cell proliferation could not explain the suppressing effect of this combination on CSC properties. Collectively, the PDE3 inhibitor synergistically potentiated the inhibitory effect of EGCG on CSCs.

### PDE3 inhibitor and a 67LR agonist in combination inhibited metastasis *in vivo*

**Figure 4:**
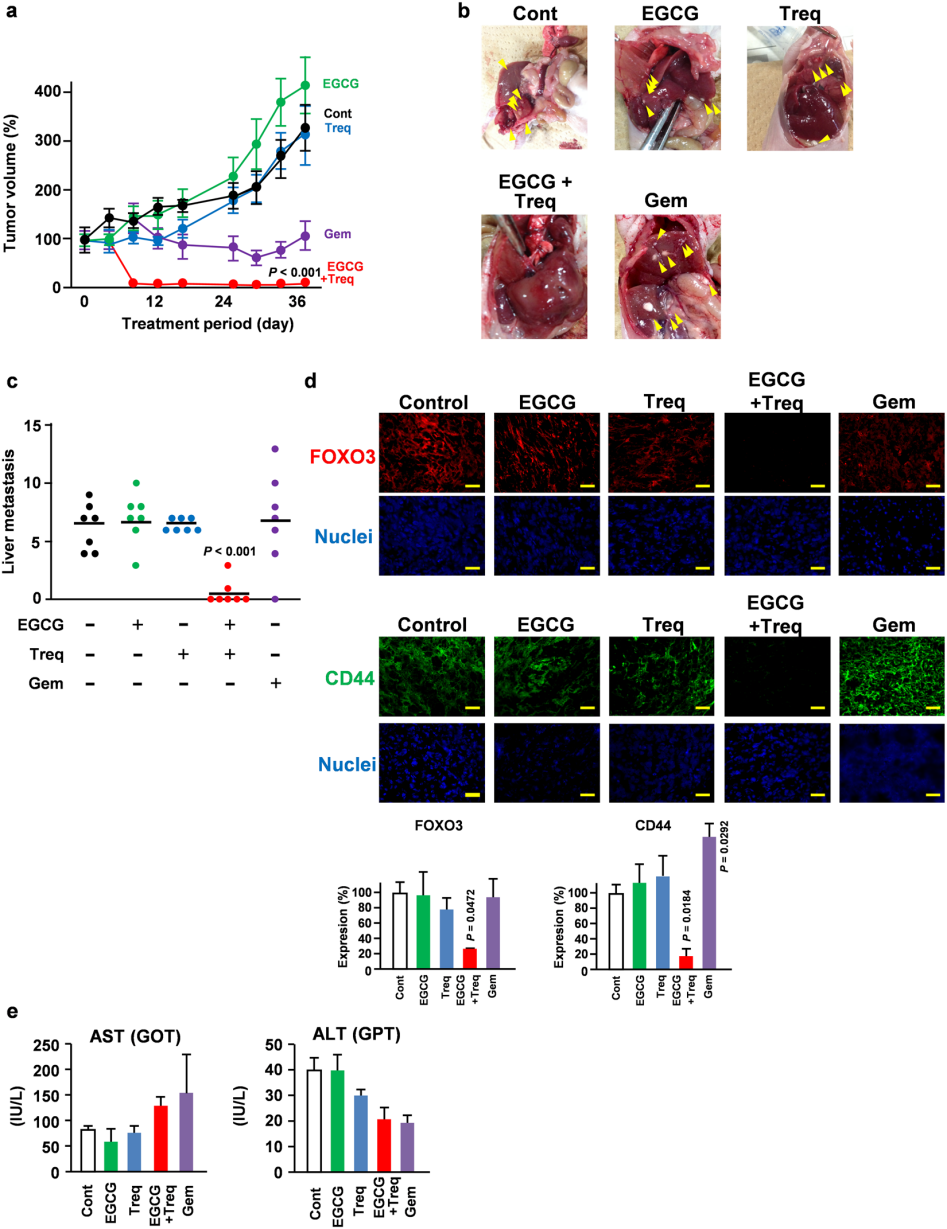
PDE3 inhibitor and 67LR agonist in combination inhibited metastasis *in vivo*. (**a**–**e**) Nude mice implanted with Panc-1 cells were injected with EGCG (10 mg/kg/2 days i.p.), trequinsin (5 mg/kg/2 days i.p.), and gemcitabine (100 mg/kg/6 days i.p.). (**a**) The tumor volume was measured (*n* = 7), (**b**,**c**) liver metastasis (*n* = 7). (**d**) Representative immunofluorescence staining for CD44 and FOXO3 on a tumor segment (×40). Scale bar: 50 μm. (**e**) AST and ALT levels in the serum were measured as an evaluation of toxicity (*n* = 7). All data are presented as the means ± SEM.

Since we previously reported that a FOXO3 knockdown drastically suppressed tumour formation and liver metastasis^14^, the inhibitory effect of EGCG and the PDE3 inhibitor in combination was evaluated based on the same xenograft models. To evaluate the effect of the combination of EGCG and the PDE3 inhibitor in a mouse model, γ-irradiated female 7-wk-old BALB/c nude mice were subcutaneously transplanted with 5 × 10^6^ Panc-1 cells per mouse. After the tumours had become palpable, the mice were injected i.p. once every two days with EGCG (10 mg/kg body weight) or trequinsin (5 mg/kg weight) and once every six days with gemcitabine (100 mg/kg weight). After 36 days of treatment, all the mice were sacrificed. Our data suggest that EGCG and the PDE3 inhibitor alone did not suppress tumour growth or metastasis (Fig. 4a–c). Gemcitabine, a conventional anti-cancer drug, suppressed the increase in tumour volume (Fig. 4a); however, it failed to suppress liver metastasis (Fig. 4b,c). In contrast, the combination of EGCG and the PDE3 inhibitor drastically suppressed both tumour formation (Fig. 4a) and liver metastasis (Fig. 4b,c). Consistent with an *in vitro* study, our immunofluorescence analysis on the tumour sections of EGCG and PDE3 inhibitor-injected mice demonstrated that the combination regimen also suppressed both FOXO3 and CD44 expression (Fig. 4d). Importantly, treatment with EGCG and the PDE3 inhibitor in combination did not affect the serum AST or ALT levels, reported to be associated with the dose-limiting toxicity of EGCG in previous clinical studies^31^ (Fig. 4e). Our results also suggested that this combination did not affect the cell growth of HUVECs (Supplementary Figure 5).

### cGMP induction could be a novel therapeutic strategy for PDAC

**Figure 5:**
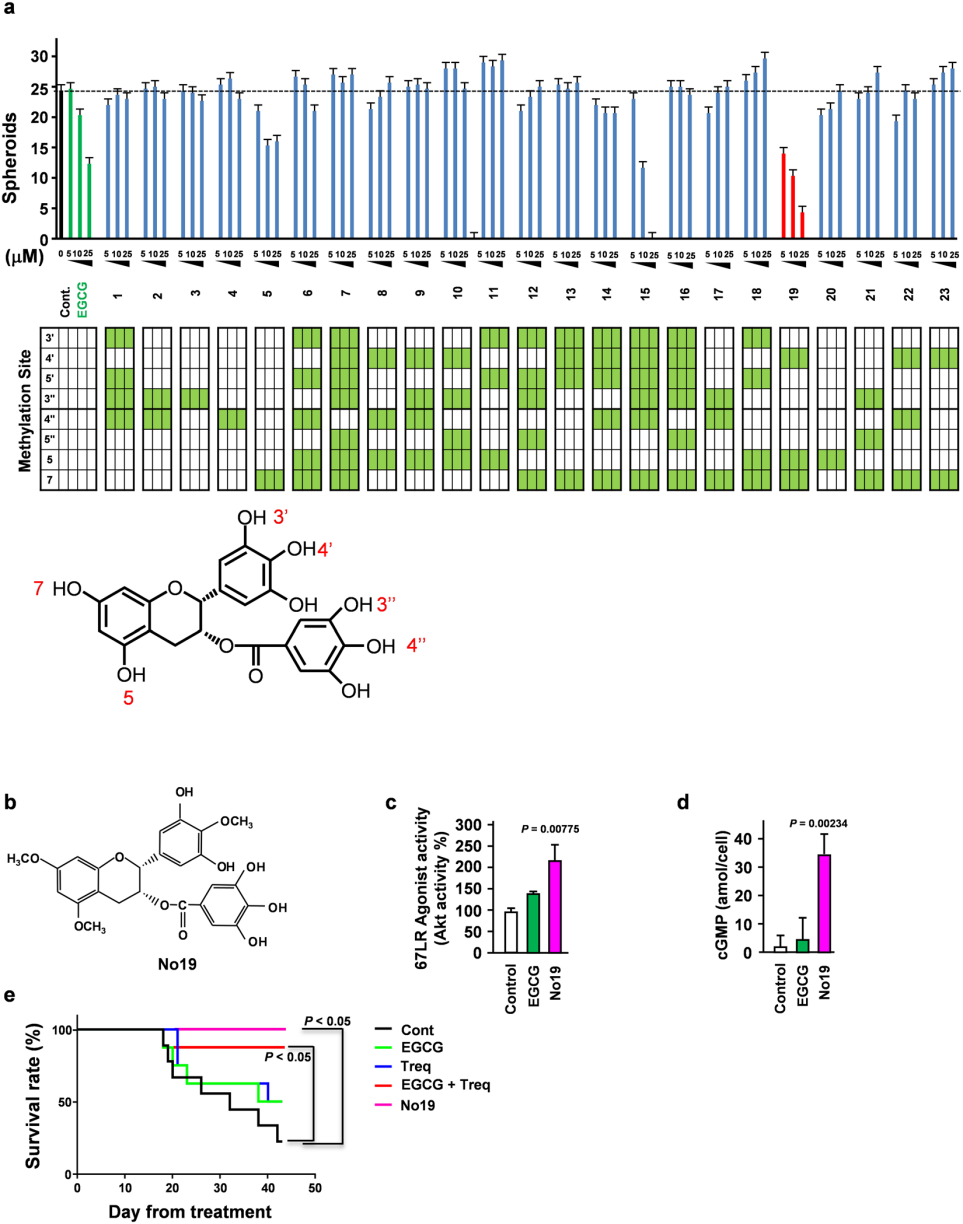
cGMP induction could be a novel therapeutic strategy for PDAC. (**a**) Panc-1 cells were treated with EGCG or its analogs (*n* = 3). (**a**,**b**) The chemical structure of the 67LR agonist No. 19 that demonstrated the strongest inhibitory effect on spheroid formation. (**c**) 67LR agonist activity determined (*n* = 3). (**d**) Intercellular cGMP levels in EGCG (5 μM) or No. 19 (5 μM) treated Panc-1 cells (*n* = 3). (**e**) BxPC-3 cell implanted mice were used as a peritoneal dissemination model and injected with EGCG (10 mg/kg/2 days i.p.), trequinsin (5 mg/kg/2 days i.p.), and No. 19 (5 mg/kg/2 days i.p.). (Control *n* = 9, the others *n* = 8). Overall survival was analyzed using a Kaplan–Meier survival model with a log-rank test.

Combinatorial chemistry is an excellent strategy used to achieve the systematic syntheses of small molecular analogs that have common core chemical structures. Solid-phase syntheses based on the chemical reactions of solid-anchored substrates, followed by the detachment of synthesized products from the solid phase, provide drastic advantages for the synthesis of several analogs based on the principal of combinatorial chemistry^35^. We previously reported that a methylated EGCG analog retains the properties of EGCG and show stable compared with EGCG^36, 37^. Our screening data based on spheroid inhibition revealed that compound No. 19 strongly suppressed spheroid formation (Fig. 5a,b) and potently activated the Akt and cGMP axis, the downstream signalling pathway induced by EGCG (Fig. 5c,d)^17^. As our data demonstrate that the combination of EGCG and a PDE3 inhibitor significantly inhibits liver metastasis (Fig. 4b,c), we hypothesized that the most applicable effect of this strategy would be the inhibition of pancreatic cancer metastasis, an important clinical characteristic of pancreatic CSCs. Peritoneal dissemination is considered to be the worst clinical scenario for patients with PDAC and is associated with a poor prognosis^38^ due to the lack of efficient clinical treatment strategies.

To assess the effect of the combination of EGCG and the PDE3 inhibitor, as well as the EGCG analog, compound No. 19 *in vivo*, female nude mice were radiated with 1.8 Gy γ. Three days after the irradiation, BxPC-3 cells were implanted to create a peritoneal dissemination mouse model. The mice were then treated with EGCG (10 mg/kg/2 days i.p.), trequinsin (5 mg/kg/2 days i.p.) and EGCG analogue No. 19 (5 mg/kg/2 days i.p.).

Accordingly, we used a peritoneal dissemination model to assess the effect of EGCG and PDE3 inhibitor in combination, as well as the EGCG analog, compound No. 19, on survival. Our data demonstrate that the combined use of EGCG and a PDE3 inhibitor in combination, as well as compound No. 19 significantly extended the survival duration in the mouse model of peritoneal dissemination (Fig. 5e). Taken together, treatment with EGCG and the PDE3 inhibitor in combination, as well as the EGCG analog exhibited a potent effect on the suppression of PDAC metastasis.

## Discussion

Potential mechanisms underlie the drug resistant phenotype of CSCs, including an enhancement of the expression of drug efflux transporters. Moreover, CSCs remain quiescent and also exhibit an enhancement of their DNA repair capacity, as well as abnormally activated developmental signaling cascades and anti-apoptotic pathways^4–7^.

Consistent with the properties of the surviving CSCs in PDAC cells, following treatment with anti-cancer agents, one study reported the drastically increased expression of CSC markers in PDAC, including CD24 (47-fold increase) and CD44 (17-fold increase)^39^. In animal study, although gemcitabine significantly inhibited the increase of tumours volume of PDAC cells with low CSC properties, the same dose of gemcitabine treatment could not suppress the increase of implanted tumours with high CSC properties^40^. The patients with low expression of both CD44^+^ and CD24^+^ had a better prognosis (an approximately 30% five-year median survival rate)^6^.

To target the CSCs of PDAC, various scientific efforts have been undertaken for decades, demonstrating that Notch, Hedgehog and Wnt/β-catenin play a crucial role in the maintenance of CSCs^7, 8^. Unfortunately, these signalling pathways are also associated with common mechanisms used by normal stem cells^41, 42^. In a recent clinical study, vismodegib, a selective inhibitor of Hedgehog signalling was used to treat patients with advanced basal-cell carcinoma; 26 of 104 of patients exhibited serious adverse events and 7 of 104 patients died due to adverse events^9^. Similarly, an inhibitor of Wnt signalling also demonstrates severe toxicity^10^. From the perspective of selective toxicity, striking molecules that are both important for normal and cancer stem cells may be a ‘double–edged sword’.

We recently reported that cGMP induction suppressed the CSC properties of PDAC through the inhibition of FOXO3^14^, previously thought to be a tumour suppressor^43^. Importantly, systemic FoxO3 KO mice did not exhibit differences in mortality for up to 48 weeks of age and any prominent cancer-prone condition^15^ while in knockout mice involving the Notch, Hedgehog and Wnt signaling pathways, such signaling deletions are lethal^44, 45^.

Here, we demonstrated that a 67LR-dependent cGMP inducer combined with inhibition of PDE3 strongly expressed in CD44^+^ cell suppressed CD44/FOXO3, the crucial master regulator for CSCs and several CSC properties of PDAC, including spheroid formation, colony formation. This combination regimen strongly suppressed liver metastasis *in vivo* and extended the survival time of mice with PDAC. We also confirmed that a synthesized EGCG analog with strong cGMP induction properties also suppressed spheroid formation of PDAC and extended the survival time of mice.

cGMP induction and PDE inhibition have been used clinically to treat patients with erectile dysfunction and ischemic heart disease^24^. Additionally, a PDE3 inhibitor was widely used as treatment of acute heart failure and cardiogenic shock^24^, as well as in patients with lung cancer for the prevention of postoperative atrial fibrillation^28^. Polyphenon E™ is a botanical drug containing 60% EGCG that has been approved for the treatment of external genital and perianal warts by the US FDA^30^. Moreover, it also exhibits anti-cancer efficacy in patients with CLL without causing severe side effects in a phase II clinical trial^31^. The safety and efficacy of this combination in patients with PDAC should be varified in future clinical trials.

A recent study suggested that FOXO3 also contributes to the persistance of leukemia-initiating cells and contribute to maintaining quiescence which may ‘fortified’ leukaemia cells from chemotherapic agents like imatinib^46^. In this context, suppression of FOXO3 drastically restores the sensitivity to chemotherapic agents in chronic myeloid leukaemia^46^. However, there is no clinically establish approach to suppress FOXO3. Our study may provide the novel approach to suppress FOXO3 by targeting 67LR because several leukemias, including chronic myeloid leukaemia exhibit significantly upregulated 67LR expression^23, 47^.

Taken together, we have shown that the 67LR-dependent cGMP inducer combined with targeting PDE3 overexpressed in CD44^+^ cells suppressed the CSC properties of PDAC. This combination inhibited liver metastasis *in vivo* and extended survival time. Our data also showed that a synthesized EGCG analog with strong cGMP induction properties inhibited the spheroid formation of PDAC and extended survival time. Our data may provide a new approach to suppress PDAC CSCs and enhance clinical outcome.

## Methods

### Study approval

All *in vivo* experiments were performed in accordance with notification (no. 6) of the Government for the welfare of animals and the Japanese law (no. 105) and were approved by the Animal Care and Use Committee (Kyushu University, Hakozaki, Fukuoka, Japan; Animal experiment number No. A26-090-4).

### Antibodies and reagents

Hoechst33342 (H3570) was purchased from Invitrogen (Carlsbad, CA, USA). Gemcitabine (G0367) was provided by the Tokyo Chemical Industry (Tokyo, Japan). EGCG (E4143), catalase (C100), and superoxide dismutase (SOD) (S5395) were obtained from Sigma-Aldrich (St. Louis, MO, USA). Trequinsin hydrochloride was obtained from Toronto Research Chemicals (Toronto, ON, Canada). EGF and FGF were purchased from BD Bioscience, (Franklin Lakes, NJ, USA). B27 were purchased from Invitrogen. Antibodies used for immunofluorescence analysis and immunoblotting consisted of an anti-FOXO3 antibody (Abcam, Cambridge, MA, USA, ab109629), anti-CV-Caspase-3 antibody (Cell Signaling Technology, #9661S), anti-β-actin antibody (Sigma-Aldrich, 061M4808), anti-CD44 antibody and anti-PDE3A (Abcam, ab112534) were used for Western blotting (Abcam, ab51037); Alexa Fluor 555-conjugated secondary antibody (Invitrogen, A21428) were used for immunofluorescence analysis. ﻿A FITC-labeled anti-CD44 antibody used in flow cytometory analysi was purchased from Miltenyi Biotec, (130-095-195 Bergisch Gladbach, Germany). Akt activity was evaluated by using assay kit obtain from Millipore (Billerica, MA, USA CBA019). Accumax was purchased from Innovative Cell Technologies (San Diego, CA, USA). The AST/ALT activity assay kit was purchased from Wako. EGCG analogs were synthesized as described previously^35^.

### Cell cultures and cell-based assays

The human pancreatic carcinoma cell lines, BxPC-3 and MIA PaCa-2, were purchased from the Japanese Collection of Research Bioresources Cell Bank. Panc-1 was obtained from the Riken Cell Bank. Primary PDAC cells were purchased from Anti-Cancer Japan (Chiba, Japan). All PDAC cells were cultured in RPMI1640 supplemented with 10% FBS supplemented and penicillin-streptomycin (Meiji Seika Pharma, Tokyo, Japan) under 5% CO_2_, 100% humidity, and at 37 °C. HUVEC cells were purchased from Kurabo, Kurashiki, Japan and cultured in EGM-2 medium.

For the colony formation assay, the cells were seeded in serum-free RPMI 1640 medium containing 1% FBS RPMI 1640 supplemented with 200 units/mL catalase and 5 units/mL superoxide dismutase at 1000 cells/well and cultured for 21 days at 37 °C and 5% CO_2_.

For the spheroid assay, PDAC cells were plated in RPMI 1640 medium supplemented with 200 units/mL catalase, 5 units/mL SOD, B27 (1:50 dilution, Invitrogen), EGF (20 ng/mL, BD Bioscience) and bFGF, (10 ng/mL, BD Bioscience) on Ultra-Low Attachment Surface 24 well-plates purchased from Corning® (#3473) at a density of 1000 cells/well, and cultured at 37 °C and 5% CO_2_ for 21 days. Akt activity was assessed as described previously^17^. cGMP was assessed using cGMP assay kit obtained from Cayman Chemicals (581021, Ann Arbor, MI, USA) in accordance with the manufacture’s protocol. QRT-PCR and FCM analysis was performed as previously described^14^. Primer information were disclosed in Supplementary Table 1.

### Immunofluorescent staining

Patients and healthy samples were purchased from US biomax and Anticancer Japan with obtaining written informed consent from all patients in accordance with the Declaration of Helsinki in those companies. Patient PDAC tissue samples and normal healthy pancreatic sample sections were deparaffinized three times in xylene and rehydrated using ethanol. Heat-induced antigen retrieval was performed at 100 °C in citrate buffer (10 mM, pH 6.0) in an oven for 10 min. The sections were incubated with 2.5% BSA-TTBS for blocking, and incubated for 7 h at 4 °C with the primary antibody. The anti-PDE3A antibody, anti-FOXO3 antibody, and anti-CV-Caspase-3 antibody were used at a 1:300 dilution and the anti-CD44 antibody was used at 1:50. The sections were stained with a secondary antibody (Alexa Fluor 555-conjugated) at a 1:100 dilution and incubated for 1 h 4 °C. The slides were rinsed and the nuclei were counterstained using Hoechst33342 (0.5 μg/mL).

### Western blotting and immunofluorescence

Panc-1 cells were seeded at a density of 2.5 × 10^4^ cells/mL in RPMI1640 medium supplemented with 10% FCS in a 5 mL dish for 24 h. The cells were treated with the indicated concentration of EGCG and PDE 3 inhibitor in PMI1640 medium supplemented with 1% FCS, 200 units/mL catalase and 5 units/mL SOD for 72 h (for CD44) and 48 h (for FOXO3). the Panc-1 cells were lysed in the lysis buffer containing 1% Triton X-100, 1 mM phenylmethanesulfonyl fluoride, 2 mg/mL aprotinin, 1 mM EDTA and 1 mM pervanadate, 30 mM Na_4_P_2_O_7_, 150 mM NaCl, 50 mM NaF in 50 mM Tris-HCl buffer at a pH of 7.5. Approximately 100 μg protein was treated with the sample buffer containing 0.001% bromophenol blue, 0.05% mercaptoethanol, 1% SDS, and 10% glycerol in a 0.1 M Tris-HCl buffer at a pH of 6.8 and boiled. The electrophoresed gels were then electroblotted onto Trans-Blot membranes (Trans-Blot) purchased from Bio-Rad Laboratories (Hercules, CA, USA). Membranes were treated with 2.5% BSA-Tween 20-TBS (TTBS) for blocking, then incubated with several antibodies diluted in 2.5% BSA-TTBS (1:3000 dilution for FOXO3 and CD44 and :10000 dilution for β-actin) overnight.

The membranes were rinsed with TTBS and treated with either anti-rabbit or anti-mouse HRP. Following the washing step, the expression levels of each protein were evaluated using ECL Ultra, TMA-6, (Mile Road, MG, USA) by following the manufacture’s protocol based on the Fusion System (Vilber-Lourmat, France).

### Xenograft murine model

Female 7-wk-old BALB/c nude mice were purchased from KBT oriental (Saga, Japan). The mice were radiated with 1.8 Gy γ-ray by using Co_60_. Three days after the irradiation, 5 × 10^6^ Panc-1 cells in RPMI1640 were subcutaneously transplanted into the right flank. After the tumors had become palpable, the mice were injected i.p. once every two days with EGCG (10 mg/kg) or Trequinsin (5 mg/kg), and once every six days with gemcitabine (100 mg/kg). Female 7-wk-old BALB/c nude were purchased from KBT oriental (Saga, Japan). The mice were radiated with 1.8 Gy γ-ray using Co_60_. Three days after the irradiation, the BxPC-3 cells were implanted into mice to establish a peritoneal dissemination model mice as described previously^48^ and injected with EGCG (10 mg/kg/2 days i.p.), trequinsin (5 mg/kg/2 days i.p.), and compound No. 19 (5 mg/kg/2 days i.p.). All animals were randomly chosen when used. Since the effect of the combination of EGCG and the PDE3 inhibitor on tumor growth *in vivo* was not predicted, we did not use a power calculation to determine an appropriate group size. Instead, we used a small group size of treated and control mice, which was demonstrated to be sufficient for all tests of statistical significance. The investigators were not blinded to the allocation during the experiments or outcome assessment.

### Statistics

All data are presented as mean the ± SEM. The correlation analysis was carried out based on a Spearman’s test. The significance of the differences between the experimental variables was determined using the one-way analysis of variance followed by Tukey’s test and unpaired Student’s t-test (two-tailed). Statistical analyses of the survival period were statistically analyzed based on the log-rank analyses of Kaplan-Meier curves. All statistical analyses were performed using GraphPad Prism 6.0 (GraphPad Software, San Diego, CA, USA). A P-value of 0.05 was considered significant.

## Electronic supplementary material

Supplemental data
