# Supplementary material for: PDE3 inhibitor and EGCG combination treatment suppress cancer stem cell properties in pancreatic ductal adenocarcinoma
Source: Sci Rep. 2017 May 15;7:1917. doi: 10.1038/s41598-017-02162-9 (PMC5432527; doi:10.1038/s41598-017-02162-9)
Supplement: Supplementary file 1 — Supplemental data [file 41598_2017_2162_MOESM1_ESM.pdf]

PDE3 inhibitor and EGCG combination treatment suppress cancer stem cell properties in pancreatic ductal adenocarcinoma

Motofumi Kumazoe[1,†], Mika Takai[1,†], Shun Hiroi[1], Chieri Takeuchi[1], Maasa Yamanouchi[2], Takashi Nojiri[3], Hiroaki Onda[3], Jaehoon Bae[1], Yuhui Huang[1], Kanako Takamatsu[1], Shuya Yamashita[1], Shuhei Yamada[1], Kenji Kangawa[3], Takashi Takahashi[4], Hiroshi Tanaka[2] and Hirofumi Tachibana[1,*]

[1]Division of Applied Biological Chemistry, Department of Bioscience and Biotechnology, Faculty of Agriculture, Kyushu University, 6-10-1 Hakozaki, Higashi-ku, Fukuoka 812-8581, Japan;

[2]Department of Applied Chemistry, Graduate School of Science and Engineering, Tokyo Institute of Technology, 2-12-1 Ookayama, Meguro, Tokyo 152-8552, Japan;

[3]Department of Biochemistry, National Cerebral and Cardiovascular Center Research Institute, 5-7-1 Fujishiro-dai, Suita-City, Osaka, 565-8565 Japan;

[4]Yokohama College of Pharmacy 601, Matana-cho, Totsuka-ku, Yokohama, Kanagawa 245-0066, Japan;

[†]These authors contributed equally to this work.

*Corresponding author

Hirofumi Tachibana

6-10-1 Hakozaki, Higashi-ku, Fukuoka 812-8581, Japan.

Tel and Fax: (+81) (92) 642-3008

E-mail: tatibana@agr.kyushu-u.ac.jp

Keywords: CSCs; PDAC; EGCG; CD44; FOXO3

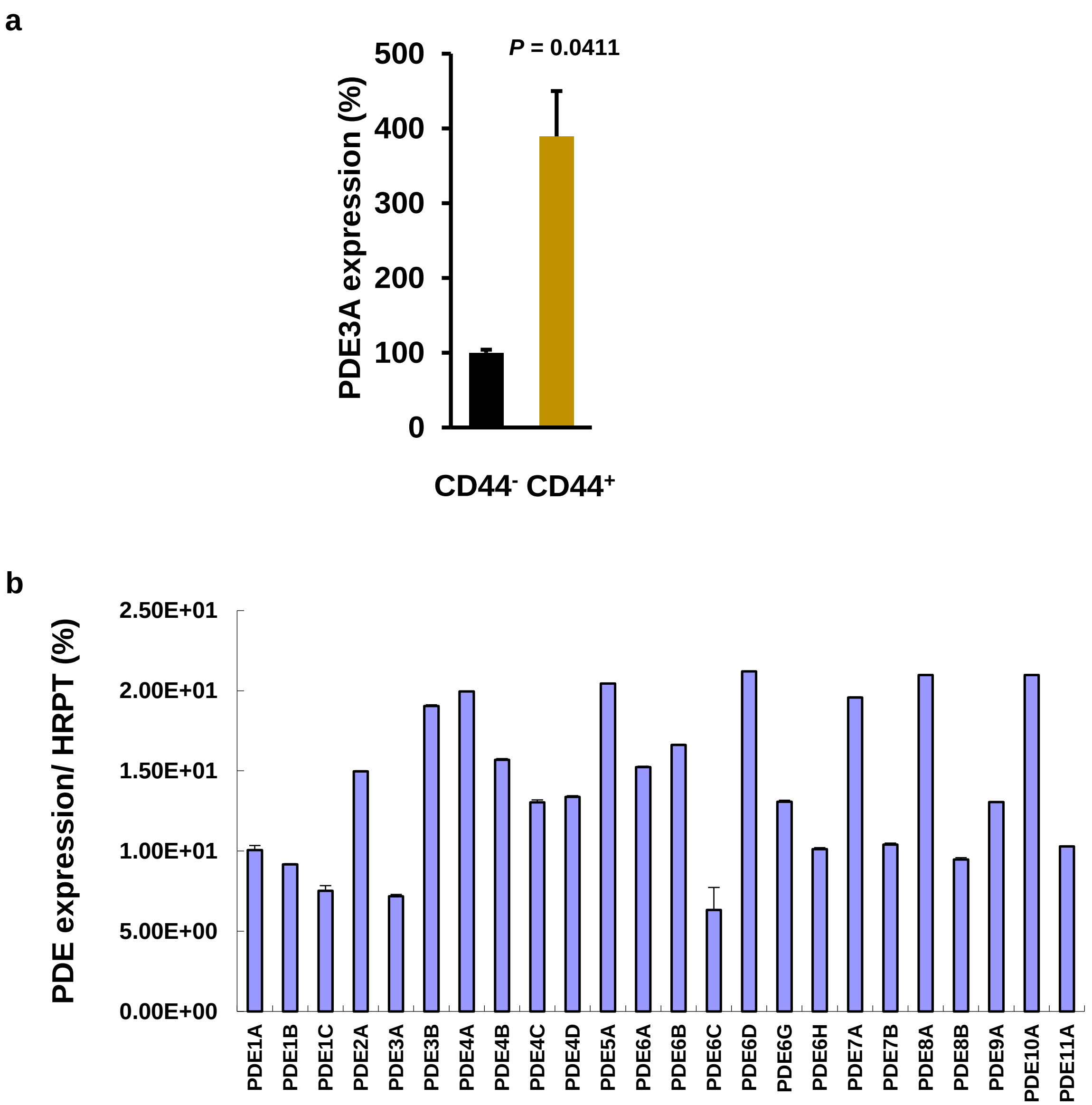

Supplementary Figure 1: Comparison of PDE3A expression between CD44$^+$ and CD44$^-$ cells.
(a) PDE3A expression was compared between CD44$^+$ and CD44$^-$ cells using FCM (n = 3). (b) PDE expression in Panc-1 cells was assessed using qRT-PCR. All data are presented as mean ± SEM.

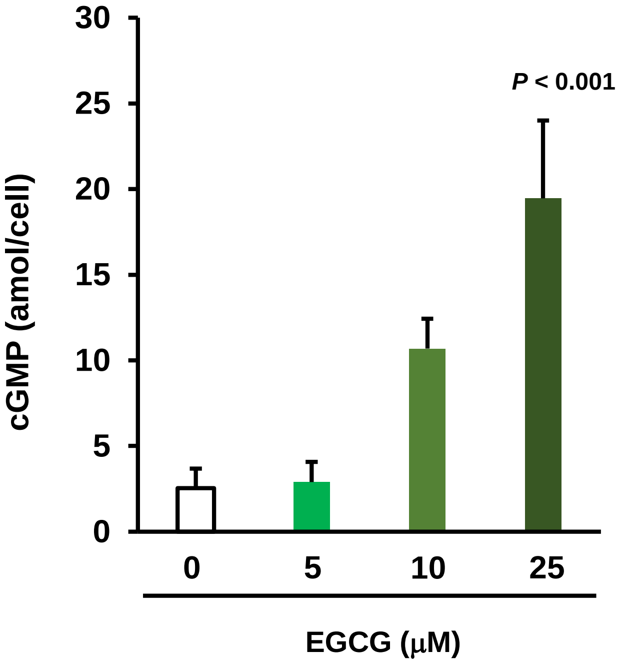

Supplementary Figure 2: Effect of high dose of EGCG on cGMP levels in Panc-1 cells.
The effect of a high dose of EGCG on cGMP levels in Panc-1 cells for 3 h (*n* = 4). All data are presented as mean ± SEM.

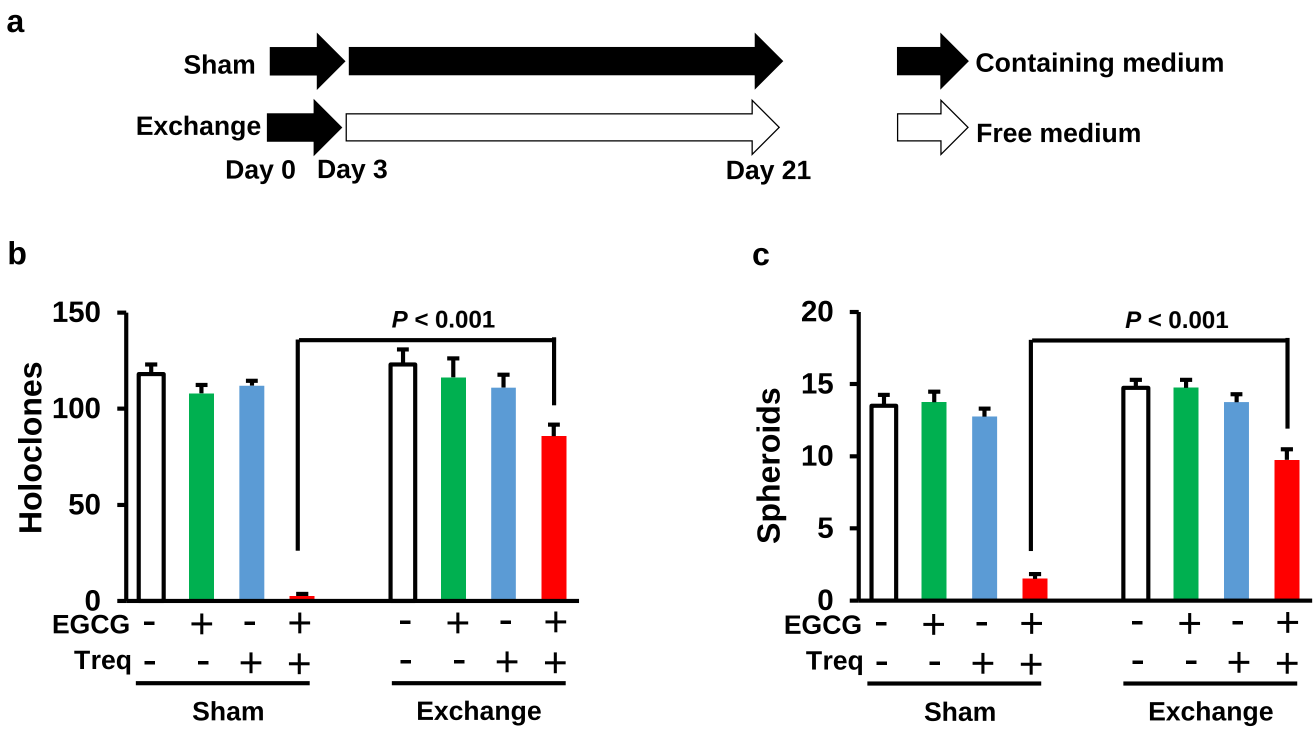

Supplementary Figure 3: Effect of short-term combined treatment of PDE3 inhibitor and EGCG. (a) Experiment schedule of the assessment of the effect of short-term combined treatment of PDE3 inhibitor and EGCG. (b) The effect of short-term combined treatment of PDE3 inhibitor (2.5 µM) and EGCG (5 µM) on colony formation (n = 3). (c) The effect of short-term combined treatment of PDE3 inhibitor (2.5 µM) and EGCG (5 µM) on spheroid formation (n = 4). All data are presented as mean ± SEM.

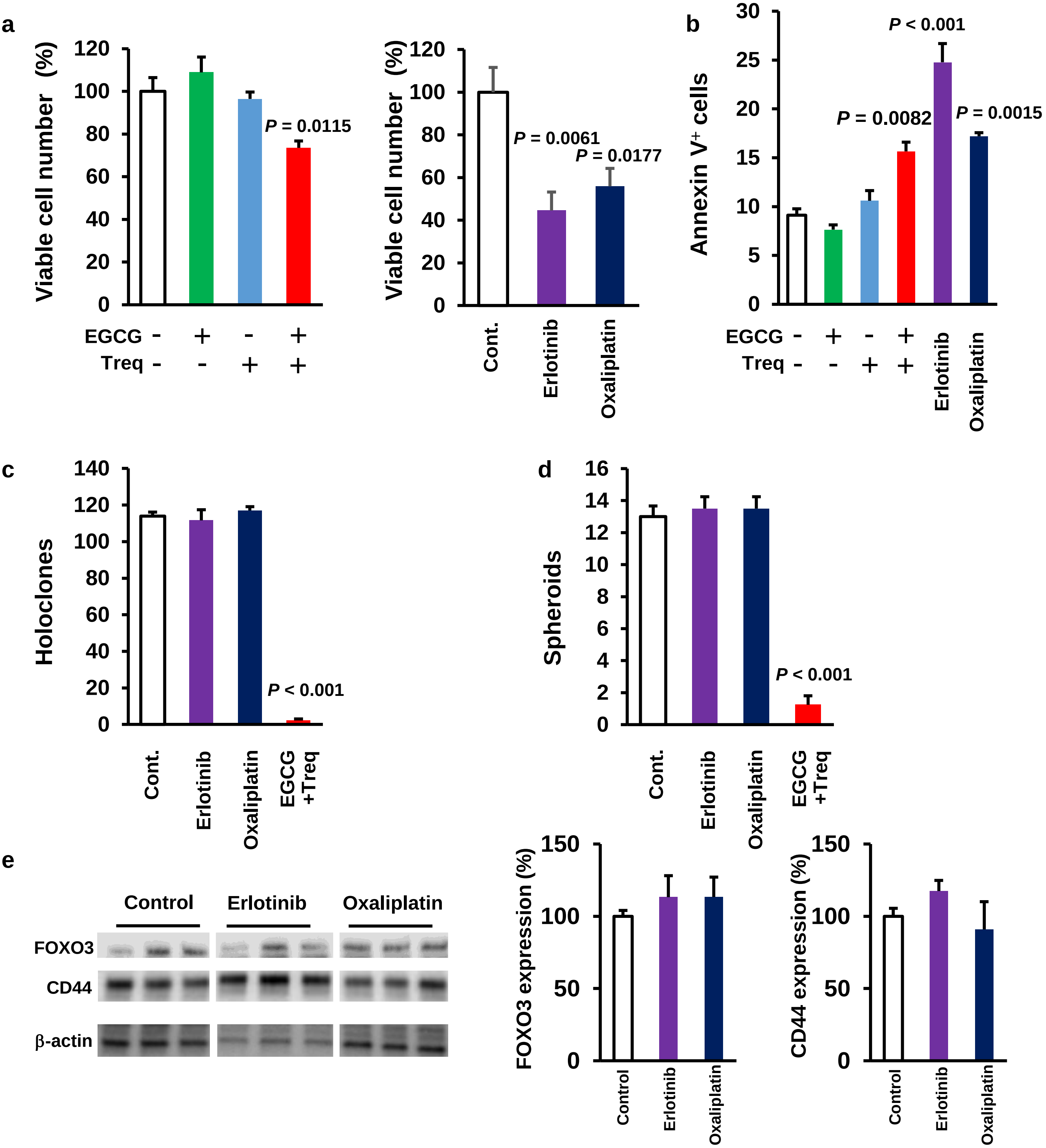

Supplementary Figure 4: Effect of anti-cancer agents on CSC properties.
(a) The effect of PDE3 inhibitor (2.5 μM), EGCG (5 μM), or erlotinib (25 μM)/oxaliplatin (25 μM) treatment for 72 h on viable cell number was determined by the ATPlite assay (*n* = 3). (b) The apoptosis-inducing effect of PDE3 inhibitor (2.5 μM), EGCG (5 μM), or erlotinib (25 μM)/oxaliplatin (25 μM) treatment for 72 h was determined by flow cytometry (*n* = 3). (c, d) The effect of erlotinib (25 μM)/oxaliplatin (25 μM) treatment for 21 days on (c) colony formation (*n* = 3) and (d) spheroid formation (*n* = 4). (e) The effect of erlotinib (25 μM) and oxaliplatin (25 μM) treatment on FOXO3 and CD44 expression in Panc-1 cells was assessed for 72 h (*n* = 3). Lanes were run on the same gel but were noncontiguous, white line. All data are presented as mean ± SEM.

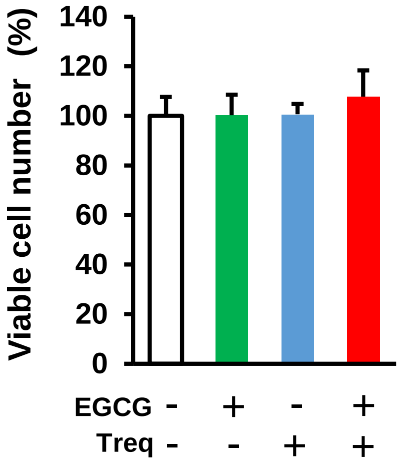

Supplementary Figure 5: Combined effects of PDE3 inhibitor and EGCG on HUVECs.
The effect of PDE3 inhibitor (2.5 μM) and EGCG (5 μM) treatment for 72 h on viable cell number in HUVECs was determined by the ATPlite assay (*n* = 3). All data are presented as mean ± SEM.

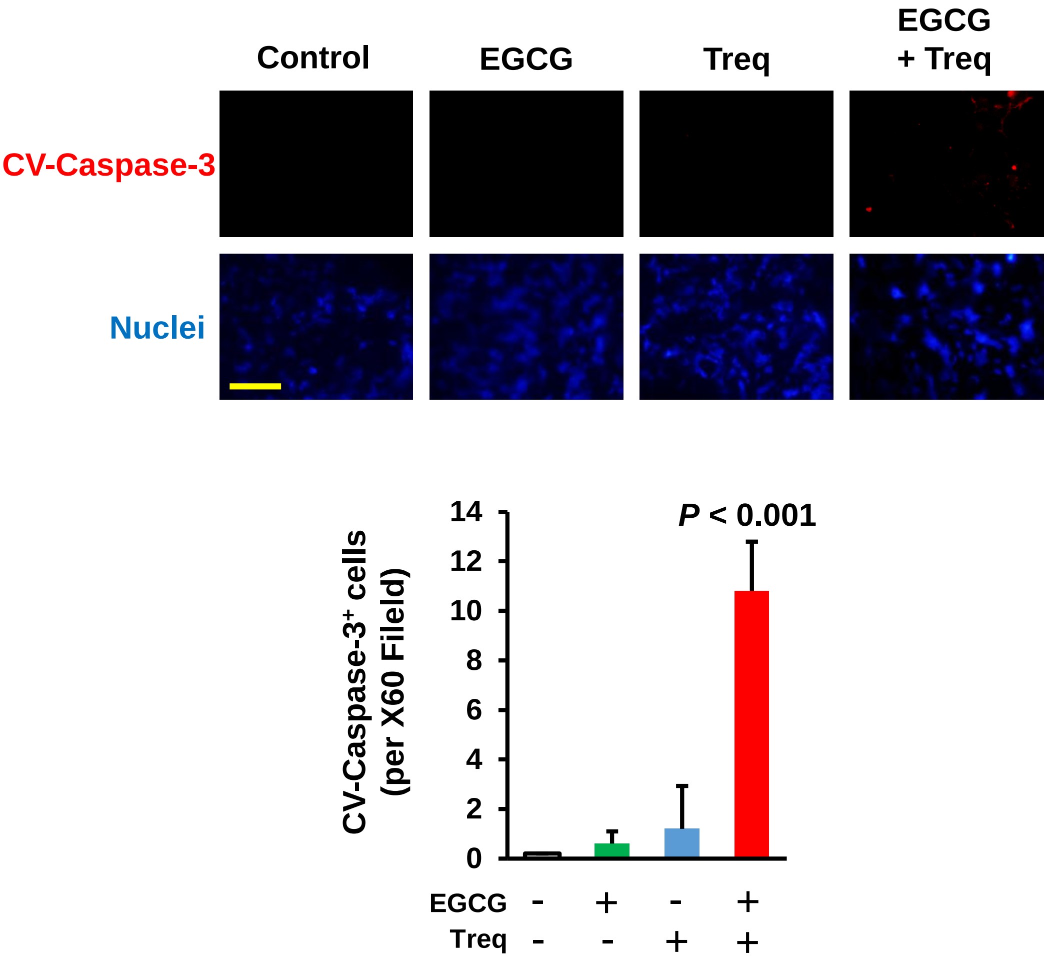

Supplementary Figure 6: Combined effects of PDE3 inhibitor and EGCG on CV-Caspase-3$^+$ cells. CV-Caspase-3$^+$ cells were evaluated by immunofluorescence analyses for 36 days. Nude mice implanted with Panc-1 cells were injected with EGCG (10 mg/kg/2 days i.p.) and trequinsin (5 mg/kg/2 days i.p.) (*n* = 5). Scale bar: 50 µm. Original magnification, ×60. All data are presented as mean ± SEM.

Supplementary Table1 :Primer list.

Gene	FORWARD（5'→3'）	REVERSE（5'→3'）
**PDE1A**	TGAAGGGATTGACAGAGC	ATGGTCCACCGATAATGC
**PDE1B**	CGCCGAGCAGAGGAGAAG	AGAACCGCAGTAGAGTAAGTGG
**PDE1C**	TGGAAGTGGGATACAGCAAGC	CTCCGTCAGCCAGTTCGC
**PDE2A**	ATCTTTGCCTTGTTTATTTCCTG	CAGCCAGCACAGATTTCG
**PDE3A**	GATGATAAATACGGATGTCTGTC	ACCGCCTGAGGAGCACTAG
**PDE3B**	TGCCTTCTTCTTCCTCACCTG	GACCACCACTGCCACACC
**PDE4A**	TTCACGGACCTGGAGATTC	TGAGGAACTGGTTGGAGAC
**PDE4B**	CAAGCCTAAACAATACAAGCATC	TGAGAATATCCAGCCACATTAAAG
**PDE4C**	CACCTGGCTGTGGGCTTC	ACTCAGTCGCTGCTTGGC
**PDE4D**	CTACTGGCTGATTTGAAGACTATG	GCTGGAGAGGCTTTGTTGG
**PDE5A**	ATCAGGAAACGGTGGGACATTTAC	CTTGTTCTCCAGCAGTGAAGTCTC
**PDE6A**	TGGCAAAGAGGACATCAAAGT	TAATCATCCATCCAGACTCATCC
**PDE6B**	GCAGAACAATAGGAAAGAGTGGA	CAGGATACAGCAGGTTGAAGA CT
**PDE6C**	AAGAATGTTTTGTCCCTGCCTA	AAGAGTGGCTTTGGTTTGGTT
**PDE6D**	AATGGTTCTTCGAGTTTGGC	AAAGTCTCACTCTGGATGTGCT
**PDE6G**	TTTAAGCAGCGACAGACCAG	ATATTGGGCCAGCTCGTG
**PDE6H**	TGAGTGACAACACTACTCTGC CT	ATGCAATTCCAGGTGGCT
**PDE7A**	AGATAGGTGCTCTGATACTAG	ATGTCTGTGTCTGGTGTC
**PDE7B**	GGCTTCTTGCTCATTTGC	CCTGTTGATGTCTGTTGC
**PDE8A**	ATGTTTGCTCGCTTTGGAATC	CAGAATGTGTAGAATTGTGGTAGG
**PDE8B**	CAAATCCCTCCGAGCACAC	CTCCATAAATCTCCTGTTGAAGC
**PDE9A**	CGTGGAATTGGAAGGACTAAAAG	GAGTCAACTTCTTGTGGTTATCC
**PDE10A**	AACTATCGGCGGGTTCCTTATC	GCGTGTGATTGTTCTGAAGTATGG
**PDE11A**	CTGCTGGGTTTCAAGACATTC	GCTTGGAAGGCATTGTTGG
